# Supra-Normal Ejection Fraction at Hospital Admission Stratifies Mortality Risk in HFpEF Patients Aged ≥ 70 Years

**DOI:** 10.3390/jcm14020426

**Published:** 2025-01-10

**Authors:** Andrea Sonaglioni, Chiara Lonati, Valentina Scime’, Gian Luigi Nicolosi, Antonino Bruno, Michele Lombardo, Sergio Harari

**Affiliations:** 1 Division of Cardiology, IRCCS MultiMedica, 20123 Milan, Italy; michele.lombardo@multimedica.it; 2 Division of Internal Medicine, IRCCS MultiMedica, 20123 Milan, Italy; chiara.lonati@multimedica.it (C.L.); valentina.scime@unimi.it (V.S.); sergio.harari@unimi.it (S.H.); 3 Department of Clinical Sciences and Community Health, Università Di Milano, 20122 Milan, Italy; 4 Division of Cardiology, Policlinico San Giorgio, 33170 Pordenone, Italy; gianluigi.nicolosi@gmail.com; 5 Laboratory of Innate Immunity, IRCCS MultiMedica, 20138 Milan, Italy; antonino.bruno@multimedica.it; 6 Laboratory of Immunology and General Pathology, Department of Biotechnology and Life Sciences, University of Insubria, 21100 Varese, Italy

**Keywords:** elderly, supra-normal ejection fraction, heart failure, HFpEF, outcome

## Abstract

**Background:** During the last few years, significant pathophysiological differences between heart failure (HF) patients with “normal” ejection fraction (EF) (50% to 64%) and those with supra-normal EF (≥65%) have been highlighted. However, these distinct EF phenotypes have been poorly investigated in elderly patients aged ≥70 y. Accordingly, the present study aimed at assessing the clinical and echocardiographic characteristics of a retrospective cohort of elderly HFpEF patients (aged ≥ 70 y), categorized on the basis of “normal” EF (50 to 64%) or “supra-normal” EF (≥65%). **Methods:** All patients aged ≥ 70 y that were discharged from our Institution with a first diagnosis of HF with preserved EF (HFpEF) between January 2020 and March 2021 entered the study. All patients underwent clinical evaluation, blood tests, and transthoracic echocardiography. The primary endpoint was “all-cause mortality”, while the secondary one was the composite “all-cause mortality or rehospitalization for all causes” over a mid-term follow-up. **Results:** A total of 200 HFpEF patients (86.4 ± 6.6 y, 70% females) were retrospectively evaluated. The “normal” EF group (*n* = 99) and the “supra-normal” EF one (*n* = 101) were separately analyzed. Compared to patients with “normal” EF, those with “supra-normal” EF were older, with greater comorbidity burden, and moderate-to-severe frailty status. The mean follow-up duration was 3.6 ± 0.3 y. During follow-up, 79 patients died, and 73 were rehospitalized. In the multivariate Cox regression analysis, age (HR 1.09, 95% CI 1.03–1.16, *p* = 0.002), EF (HR 1.08, 95% CI 1.03–1.14, *p* = 0.004), tricuspid annular plane systolic excursion (TAPSE)/systolic pulmonary artery pressure (sPAP) ratio (HR 0.14, 95% CI 0.03–0.61, *p* = 0.009), and infectious disease occurring during the baseline stay (HR 7.23, 95% CI 2.41–21.6, *p* < 0.001) were independently associated with the primary endpoint in the whole study population. EF (HR 1.04, 95% CI 1.01–1.07, *p* = 0.02) also predicted the secondary endpoint. EF ≥65% was the best cut-off to predict both endpoints. **Conclusions:** “Supra-normal” EF (≥65%) at hospital admission is independently associated with all-cause mortality and rehospitalization for all causes in elderly HFpEF patients over a mid-term follow-up.

## 1. Introduction

Heart failure with preserved ejection fraction (HFpEF) is a clinical syndrome that derives from the interaction of relevant cardiovascular risk factors and non-cardiovascular comorbidities [1].

According to the latest ESC guidelines [2], HFpEF is diagnosed in the presence of symptoms and signs of heart failure (HF), associated with a preserved ejection fraction (EF) (≥50%) on transthoracic echocardiography (TTE) and objective evidence of cardiac structural and/or functional abnormalities consistent with the presence of left ventricular (LV) diastolic dysfunction and raised left ventricular filling pressures (LVFP).

The prevalence of HFpEF is increasing worldwide, accounting for more than 50% of HF cases [3]. Due to the increased burden of non-cardiovascular comorbidities in HFpEF patients, these individuals have a greater risk of all-cause hospitalization and emergency visits and are more likely to experience non-cardiac death, in comparison to HF patients with reduced EF [3].

During the last few years, a new category of HFpEF characterized by “supra-normal” EF (≥65%) [4,5], named HF with “supra-normal” ejection fraction (HFsnEF), has been proposed. Some studies have highlighted significant pathophysiological differences between HF patients with EF 50% to 64% (the “normal” EF phenotype) in comparison to those with EF ≥ 65% (the “supra-normal” EF phenotype) in both chronic [6,7] and acute [8,9] HF. However, the majority of studies that evaluated the prognostic role of a “supra-normal” EF included predominantly middle-aged participants, and as far as we know, no previous study has specifically focused on elderly HFpEF patients. As a consequence of the increasing population ageing worldwide, the age limit for the elderly has recently been shifted from 65 y to over 70 y [10]. Literature data suggest that HFpEF patients aged ≥ 70 y are increasingly encountered in clinical practice [11,12]. Accordingly, the present study aimed at assessing the clinical and echocardiographic characteristics of a retrospective cohort of elderly HFpEF patients (aged ≥ 70 y), categorized on the basis of “normal” EF (50 to 64%) or “supra-normal” EF (≥65%), and to determine the independent prognostic indicators of “all-cause mortality” and “rehospitalizations for all causes” over a mid-term follow-up in the whole study population.

## 2. Materials and Methods

### 2.1. Study Population

All elderly patients aged ≥ 70 y discharged from the Internal Medicine Division of our Institution, with a first diagnosis of HFpEF, between 1st January 2020 and 27th March 2021, were included in this retrospective observational study. The study group was selected from a larger population of HF patients, previously analyzed in research that aimed to investigate the prevalence and clinical outcome of the main hemodynamic HF phenotypes [13].

As recommended by the 2021 ESC guidelines [2], HFpEF diagnosis was formulated in the presence of symptoms (dyspnea, fatigue, or decreased exercise capacity); signs (edema or rales on chest auscultation); preserved EF (≥50%) at the TTE examination performed at admission to the Internal Medicine Division; elevated levels of natriuretic peptides [N-terminal pro-brain natriuretic peptide (NT-proBNP) ≥ 125 pg/mL)]; and at least one of the following: relevant structural heart disease (LV hypertrophy or left atrial enlargement) and/or diastolic dysfunction.

Inclusion criteria were all HFpEF patients (EF ≥ 50%) hospitalized at the Internal Medicine Division of our Institution between 1st January 2020 and 27th March 2021, in whom conventional TTE was performed during their hospital stay.

Exclusion criteria were heart failure with reduced ejection fraction (HFrEF) (EF ≤ 40%), heart failure with mildly reduced ejection fraction (HFmrEF) (EF 41–49%), age < 70 y, hemodynamic instability, HF patients in whom conventional TTE was not performed during hospital stay, poor echocardiographic windows, and COVID-19 patients.

HFpEF patients were divided in two major groups, according to EF value obtained at TTE performed at hospital admission: (1) HFpEF patients with EF 50 to 64% (the “normal” EF group); (2) HFpEF patients with EF ≥ 65% (the “supra-normal” EF group). This cut-off was derived from previous population studies [5,14] that analyzed the prognostic role of “supra-normal” EF (≥65%) over a mid-term follow-up.

HFpEF patients were further classified as affected by one of the following clinical subtypes of HF, based on predominant etiology and pathophysiology: (1) HF due to acute/chronic coronary artery disease (CAD); (2) HF due to acute/chronic valvular heart disease (VHD); (3) HF due to hypertensive cardiomyopathy; and (4) HF due to acute/chronic pulmonary hypertension [2].

Data collected from patients’ hospital medical charts are summarized in Supplementary Table S1.

All procedures were in accordance with the ethical standards of our Institutional Research Committee and with the 1964 Helsinki Declaration and its later amendments or comparable ethical standards. The study protocol was approved by the local Ethics Committee (reference number 464.2021).

### 2.2. Clinical Prognostic Scores

For each HFpEF patient included in the present study, the following clinical scores were calculated: 1) H2FPEF score [15], with the scores < 2 and ≥6 reflecting low and high likelihoods of HFpEF, respectively; 2) Charlson Comorbidity Index (CCI) [16], where a CCI score ≥5 is indicative of severe comorbidity burden; 3) AGILE score [17], which allows for the quantification of the frailty degree of HFpEF patients as follows: not frail (score 0) and light (score 1–3), moderate (score 4–7), or severe (score 8–10) frailty.

### 2.3. Conventional Transthoracic Echocardiography

All TTE examinations were performed by the same cardiologist (A.S.) within 48 h after hospital admission, using a Philips Sparq ultrasound machine (Philips, Andover, MA, USA) with a 2.5 MHz transducer.

EF was estimated with the biplane modified Simpson’s method [18]. Doppler measurements included the E/A ratio and E/average e’ ratio, the latter as an index of LVFP [19]. Systolic pulmonary artery pressure (sPAP) was derived by the modified Bernoulli equation, where sPAP = 4 × [tricuspid regurgitation velocity (TRV)] ^2^ + right atrial pressure [20]. The latter was estimated from the IVC diameter and collapsibility. Finally, the TAPSE/sPAP ratio was measured as the noninvasive index of right ventricular (RV)/pulmonary artery (PA) coupling [21].

The degree of valvulopathy was assessed, according to the AHA/ACC recommendations for the management of patients with VHD [22].

### 2.4. Endpoint Definition

The primary objective of the study was to identify the independent predictors of “all-cause mortality” (primary endpoint) in the whole population of HFpEF patients, over a medium-term follow-up. The secondary aim of the study was to evaluate the independent predictors of the composite “all-cause mortality or rehospitalization for all causes” (secondary endpoint) in the same study group.

Causes of death and rehospitalization for each HFpEF patient were collected and ascertained by the same physician (V.S.) by accessing medical records available in the hospital archive and/or from telephone interviews.

### 2.5. Statistical Analysis

HFpEF patients enrolled in the study were stratified in two major groups: 1) HFpEF patients with EF 50 to 64% (the “normal” EF group) and 2) HFpEF patients with EF ≥65% (the “supra-normal” EF group).

Each continuous variable was checked through the Shapiro–Wilk test, and all data were determined to be normally distributed. Accordingly, continuous data were summarized as mean ± standard deviation, while categorical data were presented as numbers (percentage). Continuous variables were compared using a two-sample independent t test; whereas, categorical parameters were compared using the Chi-squared test or the Fisher’s exact test.

Univariate and multivariate Cox regression analyses were performed to identify the independent predictors of the primary and secondary endpoints in the whole study population, over a mid-term follow-up.

The receiver operating characteristics (ROC) curve analysis was performed to establish the sensitivity and the specificity of the statistically significant continuous variables in the Cox multivariate analysis for predicting both outcomes. The area under curve (AUC) was estimated.

The survival curves and the event-free survival curves of the variables statistically significant in the Cox multivariate analysis were estimated using the Kaplan–Meier method, and the survival curves were compared using the log-rank test.

The intraclass correlation coefficient (ICC) with its 95% confidence interval (CI) was used as a statistical method for assessing intra- and inter-observer measurement variability in the assessment of EF.

Statistical analysis was performed with SPSS version 28 (SPSS Inc., Chicago, IL, USA), with two-tailed *p*-values < 0.05 deemed statistically significant.

## 3. Results

### 3.1. Baseline Clinical Characteristics

During the study period, 230 HFpEF patients aged ≥ 70 y were selected from the original study population [13]; among them, 15 COVID-19 patients were excluded, 10 patients were excluded due to poor echocardiographic window, and 5 due to lack of complete data. Therefore, a total of 200 HFpEF patients (mean age 86.4 ± 6.6 y) were retrospectively evaluated. The “normal” EF (50 to 64%) group (*n* = 99) and the “supra-normal” EF (≥65%) group (*n* = 101) were separately analyzed.

Table 1 summarizes the main demographics and clinical parameters recorded in the whole study population and in the two EF groups at hospital admission.

Females represented the great majority of the whole study population (70%), with similar prevalence in both EF groups.

More than half of HFpEF patients were aged 85 y and older (“oldest-old”). The “oldest-old” patients were significantly more prevalent in the “supra-normal” EF group than in the “normal” EF one.

The HFpEF patients included showed a high prevalence of hypertension (detected in 76% of patients) and a moderate prevalence of dyslipidemia (observed in approximately half of patients); whereas, type 2 diabetes mellitus and smoking history were much less commonly found.

The most frequent comorbidities detected in our study population were anemia and chronic kidney disease (CKD), observed in approximately two-thirds of patients. Cognitive impairment was found in one-third of patients, while chronic obstructive pulmonary disease, obstructive sleep apnea syndrome, a history of CAD and/or stroke, and peripheral arteriopathy were observed in approximately one-fifth of patients. Arterial hypertension, CKD, and hypothyroidism were more frequently diagnosed in HFpEF patients with “supra-normal” EF; whereas, a history of CAD was more commonly detected in those with “normal” EF.

The average value of the H2FPEF score detected in our study population at hospital admission (5.1 ± 1.9) was compatible with an HFpEF diagnostic probability of >80%. Moreover, our study population showed an elevated burden of comorbidity and frailty, as assessed by CCI and AGILE scores, respectively. Both CCI and AGILE scores were significantly higher in the “supra-normal” EF group than in the “normal” EF one.

On physical examination, blood pressure values and heart rate were normal; lower extremity edema was observed in only 19.5% of HFpEF patients; a body temperature ≥ 37.5° was found in 35.5% of cases. Chest X-rays revealed pulmonary congestion and pneumonia in 34% and 24% of HFpEF patients, respectively. Compared to the “normal” EF group, the “supra-normal” EF one showed significantly lower systolic and diastolic blood pressure values and a significantly higher heart rate, compatible with hypovolemia. Accordingly, fever and pneumonia were much more commonly observed among HFpEF patients with “supra-normal” EF, while radiographic signs of pulmonary congestion were much more frequently diagnosed in HFpEF patients with EF between 50% and 64%.

On blood tests performed at the admission to the Internal Medicine Division, the whole group of HFpEF patients was diagnosed with a moderate increase in serum levels of fasting glucose, uric acid, C-reactive protein (CRP), and NT-proBNP, a mild increase in serum levels of high-sensitivity troponin, and finally, a moderate reduction in serum levels of high-density lipoprotein (HDL) cholesterol. Compared to HFpEF patients with “normal” EF, those with “supra-normal” EF showed a greater impairment in renal function, as expressed by serum creatinine and estimated glomerular filtration rate value, significantly higher serum levels of thyroid-stimulating hormone, CRP, and white blood cells, and a significantly lower partial pressure of oxygen in the arterial blood (PaO2).

### 3.2. Transthoracic Echocardiography Findings

Concerning conventional TTE parameters, the whole group of HFpEF patients was found with normal biventricular cavity sizes and normal biventricular systolic function, as assessed by LVEF and TAPSE, respectively; a first degree of diastolic dysfunction was the most common LV filling pattern observed among HFpEF patients, being detected in 46% of patients; LVFPs, as assessed by the average E/e’ ratio, were in the so-called “grey zone” between 8 and 13; whereas, LA size was moderately increased. TTE showed moderate-to-severe mitral regurgitation and tricuspid regurgitation in approximately one-third of HFpEF patients, while aortic valvulopathies were less commonly detected. An analysis of pulmonary hemodynamics revealed a moderate increase in sPAP and a concomitant impairment in the TAPSE/sPAP ratio, indicative of RV/PA uncoupling. In comparison to the “normal” EF group, the “supra-normal” EF one was found with significantly smaller biventricular cavity sizes, significantly greater RWT, higher LVEF, and lower LVFP, thus suggesting a hypovolemic status. In addition, a systolic intraventricular (IV) pressure gradient ≥15 mmHg, obtained by continuous-wave Doppler, and generated by LV hypercontractility, was detected in approximately two-thirds of patients with “supra-normal” EF. With regards to pulmonary hemodynamics, a concomitant impairment in the TAPSE/sPAP ratio was significantly more enhanced among HFpEF patients with “supra-normal” EF than in those with “normal” EF. On the other hand, HFpEF patients with “normal” EF were more commonly diagnosed with larger cardiac chamber cavity sizes, higher LVFP, and increased prevalence of hemodynamically relevant mitral regurgitation, thus indicating congestive status (Table 2).

### 3.3. HFpEF Characteristics and Hospitalization Data

A detailed analysis of HF characteristics and hospitalization parameters recorded in the whole population of HFpEF patients and in the two EF groups is reported in Table 3.

At hospital admission, 50% of HFpEF patients were in NYHA Class III and the remaining 50% of the total were in NYHA Class IV. Approximately two-thirds of HFpEF patients with “supra-normal” EF (60.4% of total) were in NYHA Class IV; whereas, 60.6% of those with “normal” EF were in NYHA Class III.

Hypertensive cardiomyopathy was the most common cause of HF in the whole study group (being detected in 76% of cases), followed by acute/chronic VHD and acute/chronic pulmonary hypertension, both detected in approximately half of patients. Hypertensive cardiomyopathy was the prevalent etiology of HFpEF with “supra-normal” EF, while acute/chronic VHD and, to a lesser extent, acute/chronic CAD were the most frequent HF causes in patients with “normal” EF.

HFpEF patients with “supra-normal” EF were commonly hospitalized for an infectious disease of pulmonary (more than 50% of cases) or non-pulmonary origin (approximately 25% of cases). A large number of non-cardiac diseases, not rarely concomitant, such as severe anemia, severe CKD, gastro-intestinal disorders, cancers, electrolyte disorders, and neurological disorders, represented the main reasons for hospitalizations among the “supra-normal” EF group. Conversely, the “normal” EF group was more frequently hospitalized due to congestive HF, with a lower prevalence of relevant comorbidities.

At hospital discharge, more than half of HFpEF patients were prescribed with beta blockers, particularly those with “supra-normal” EF, and less than half of total with loop diuretics, especially those with “normal” EF; whereas, the remaining cardioprotective drugs were less frequently prescribed in both groups of patients.

Finally, the average length of hospital stay was 11.7 ± 5.5 days, with no statistically significant differences between the two groups of HFpEF patients.

### 3.4. Survival Analysis

The mean follow-up period was 3.6 ± 0.3 y. During follow-up, 79 patients died, and 73 were rehospitalized. In total, 87.3% of HFpEF patients died due to non-cardiovascular causes; whereas, cardiovascular causes of death were detected in the minority of HFpEF patients. The great majority of deaths (91.1% of total) were recorded among HFpEF patients with “supra-normal” EF. Rehospitalization for all causes was observed in 59.4% of the HFpEF patients with “supra-normal” EF and 13.1% of the HFpEF patients with “normal” EF. Rehospitalization due to non-cardiovascular (CV) causes was much more commonly detected among the “supra-normal” EF group; whereas, the percentage of HFpEF patients rehospitalized due to CV causes was similar in the two EF groups. A detailed temporal analysis of the rates of mortality and rehospitalization due to non-CV or CV causes over follow-up period recorded in the whole study population and in the two EF groups is summarized in Table 4.

In the univariate Cox regression analysis (Table 5), the following variables were significantly correlated with the primary endpoint: age, female sex, eGFR, AGILE score, LVEF, TAPSE/sPAP ratio, and infectious disease during baseline stay.

In the multivariate Cox regression analysis, age (HR 1.09, 95% CI 1.03–1.16, *p* = 0.002), LVEF (HR 1.08, 95% CI 1.03–1.14, *p* = 0.004), TAPSE/sPAP ratio (HR 0.14, 95% CI 0.03–0.61, *p* = 0.009), and infectious disease occurring during the baseline stay (HR 7.23, 95% CI 2.41–21.6, *p* < 0.001) maintained statistical significance. The ROC curve analysis highlighted that age ≥ 85 y (AUC = 0.82; 95% CI 0.76–0.88), LVEF ≥ 65% (AUC = 0.92; 95% CI 0.88–0.96), and TAPSE/sPAP ratio ≤ 0.55 mm/mmHg (AUC = 0.93; 95% CI 0.89–0.97) were the cut-off values with the highest sensitivity and specificity for predicting “all-cause mortality” in the whole study group.

**Figure 1:**
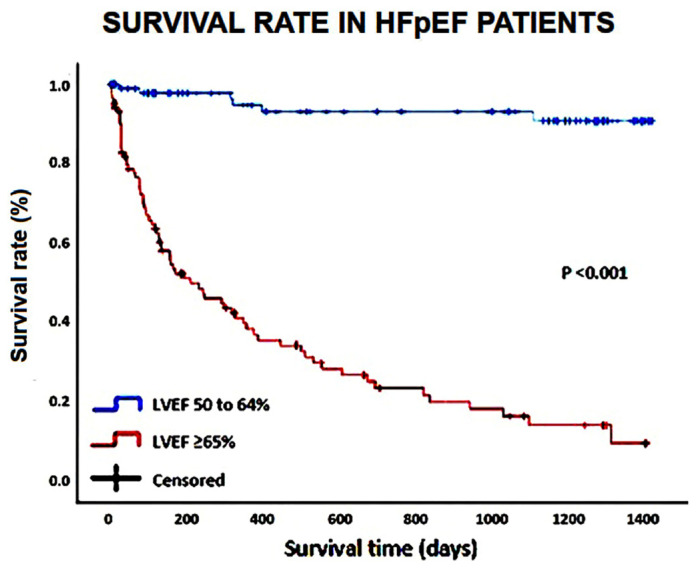
Kaplan–Meier survival curves drawn to compare the rates of “all-cause mortality” in HFpEF patients, categorized according to LVEF (50 to 64% and ≥65%, respectively). HpEF, heart failure with preserved ejection fraction; LVEF, left ventricular ejection fraction.

The Kaplan–Meier survival curves drawn for comparing the rates of “all-cause mortality” in HFpEF patients categorized according to LVEF (50 to 64 and ≥65%, respectively) are illustrated in Figure 1.

In the multivariate Cox regression analysis performed for identifying the independent predictors of the composite “all-cause mortality or rehospitalizations for all causes”, only LVEF (HR 1.04, 95% CI 1.01–1.07, *p* = 0.02) was independently associated with the secondary endpoint (Table 6).

LVEF ≥ 65% (AUC = 0.78; 95% CI 0.72–0.85) showed 64% sensitivity and 99% specificity for predicting the secondary endpoint.

**Figure 2:**
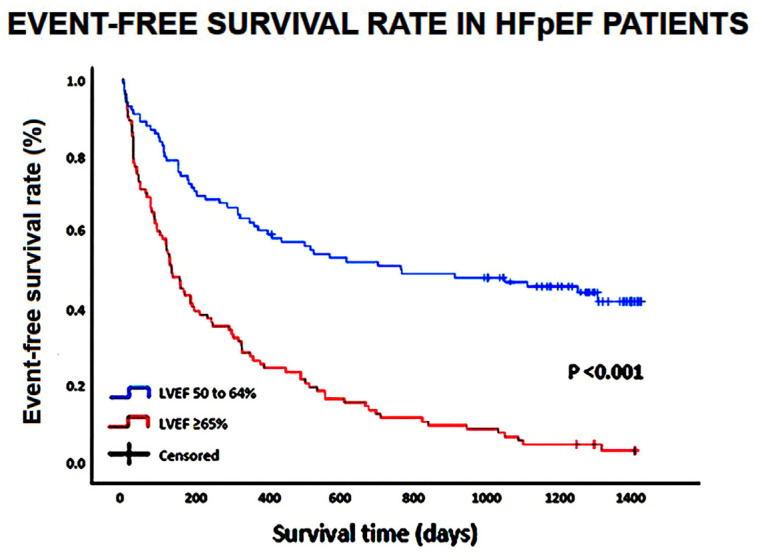
Kaplan–Meier survival curves drawn to compare the rates of the composite of “all-cause mortality or rehospitalization for all causes” in HFpEF patients, categorized according to LVEF (50 to 64% and ≥65%, respectively). HFpEF, heart failure with preserved ejection fraction; LVEF, left ventricular ejection fraction.

The Kaplan–Meier survival curves drawn for comparing the rates of the composite “all-cause mortality or rehospitalizations for all causes” in HFpEF patients categorized according to LVEF (50 to 64 and ≥65%, respectively) are depicted in Figure 2.

### 3.5. Measurement Variability

A detailed intra- and inter-observer variability analysis of EF assessment was conducted in a group of 15 randomly selected HFpEF patients. The intra- and inter-observer agreement between the raters, expressed as ICCs, was 0.92 (95% CI 0.77–0.97) and 0.81 (95% CI 0.51–0.93), respectively.

## 4. Discussion

The present study identified two opposite phenotypes of elderly HFpEF patients: (1) those with “normal” EF (between 50 and 64%), characterized by the increased prevalence of radiographic and echocardiographic congestive signs; (2) those with “supra-normal” EF (≥65%), who were older, predominantly females, with high comorbidity burden and severe frailty status, commonly hospitalized for infectious diseases and frequently found with clinical and echocardiographic signs of reduced preload due to dehydration and hypovolemia. The “supra-normal” EF phenotype showed a ten-fold higher mortality rate and a two-fold higher prevalence of the composite of “all-cause mortality or rehospitalizations for all causes” than the “normal” EF one, over a mid-term follow-up. Age, EF, and infectious disorder were linearly correlated with “all-cause mortality” in the whole study group; whereas, the TAPSE/sPAP ratio showed a strong inverse correlation with the primary endpoint. Among HFpEF patients, those aged ≥ 85 y, hospitalized for an infectious disorder, and diagnosed with “supra-normal” EF (≥65%) and TAPSE/sPAP ratio ≤ 0.55 mm/mmHg on TTE examination at hospital admission had the worst prognosis. On the other hand, HFpEF patients with EF 50 to 64% hospitalized due to congestive HF, aged < 85 y, without infection, and without RV/PA uncoupling showed a less complicated clinical course and a more favorable outcome.

During the last two decades, a few studies have evaluated the prognostic role and the clinical implications of a “supra-normal” EF (≥65%) in different study populations [14,23,24]. A U-shaped relationship between EF and mortality was demonstrated among women undergoing routine echocardiography for suspected or established cardiovascular disease [23], in elderly female patients (aged > 65 years) with acute coronary syndromes [24], and in a large and heterogeneous clinical cohort of patients referred for echocardiography [14]. Similar findings have been reported for both chronic [6,7] and acute [8,9] HF patients. These studies attributed the genesis of HFsnEF to a number of pathophysiological mechanisms, such as abnormal LV systolic stiffening, small LV cavity size, increased arterial elastance, and severe diastolic dysfunction [25]. These factors may synergically affect LV filling, with a secondary reduction in stroke volume and cardiac output. The apparent paradox of rising EF in the face of a falling stroke volume was primarily ascribed to the overall reduction in LV cavity volumes [26].

Consistent with the studies conducted on both chronic [6,7] and acute [8,9] HF patients, our findings confirmed that a “supra-normal” EF was a negative prognostic factor over a mid-term follow-up period. Differently from these studies that evaluated a larger population of middle-aged HF patients, the present retrospective analysis was conducted on a cohort of elderly HFpEF patients with a consistent number of individuals aged ≥85 y. The “supra-normal EF” was more commonly detected in the context of an infectious disease related to pulmonary or, more rarely, to non-pulmonary infections. The infective process associated with elevated body temperatures and fluid loss was probably responsible for dehydration, hypovolemia, and a reduction in preload, thus causing a hypercontractile response. In addition, dehydration determined an increase in sympathetic activity, as previously shown [27]. The frequent detection of an IV pressure gradient and the expression of the instantaneous ejection intraventricular pressure difference between the LV basis and apex, which reached its peak early during end-systole [28], confirmed the hyperdynamic LV systolic function. The hypovolemic status secondary to dehydration was associated with increased serum levels of NT-proBNP due to the concomitant presence of renal impairment in HFpEF patients with “supra-normal” EF.

Finally, the RV/PA uncoupling highlighted by TTE in these patients was likely related to acute or persistent hypoxia in the setting of a respiratory distress syndrome and/or infectious disorder. In our findings, a TAPSE/sPAP ratio ≤ 0.55 mm/mmHg was independently associated to an increased risk of all-cause mortality in the whole cohort of HFpEF patients. This parameter represents a simple echocardiographic index of RV systolic function, “matched” to an index of RV afterload [21]. During the last few years, several studies have demonstrated that a reduced TAPSE/sPAP ratio is a strong predictor of mortality and recurrent hospitalizations in both HFpEF [29,30,31,32] and HFrEF [33] patients. Accordingly, our results were in alignment with the literature data.

Differently from other studies [9], in our population, we did not observe greater LA dimensions in “supra-normal” EF patients than in “normal” EF ones. This could be unexpected, being LA dilatation strongly correlated with hypertensive and kidney disease, but it could be related to the markedly hypovolemic status of the “supra-normal” EF group or to the fact that our echocardiographic data were not indexed to body surface area (BSA). Additionally, as recently proposed by Popovic et al. [34], despite elevated LVFP, HF patients with EF ≥ 65% may have a form of cardiac contracture, influencing both the left ventricle and left atrium.

With regards to HFpEF patients with EF 50 to 64% included in the present study, our results suggest that these patients have a number of clinical characteristics similar to those of HFmrEF patients, such as less advanced age, a more frequent history of CAD, and a reduced burden of comorbidity/frailty, as previously reported by our study group [35]. In light of these evidence, they could represent a category of HF patients who have recovered from previous HFmrEF, thus confirming that HFpEF patients represent a heterogenous and dynamic group of patients, rather than a unique HF subtype.

Concerning the medical treatment of the HFpEF patients included in the present study, the majority of patients were treated with loop diuretics and beta blockers; whereas, other cardioprotective drugs, such as angiotensin-converting enzyme inhibitors (ACEIs), angiotensin receptor blockers (ARBs), and statins, were less commonly used. The undertreatment with ACEIs/ARBs in HFpEF patients was likely related to the lack of benefit of these drugs in HF patients with EF ≥ 65%, as indicated by the literature data [36,37]. The separate analysis of the two groups of HFpEF patients revealed that loop diuretics were much more frequently administered to the “normal” EF group; whereas, beta blockers to the “supra-normal” EF one. This medical choice was probably conditioned by the congestive status or the hypovolemic/hyperdynamic status, respectively, observed in the two distinct groups of HFpEF patients. Finally, no HFpEF patient included in the present study was treated with sodium/glucose cotransporter 2 (SGLT2) inhibitors.

As already underlined [38,39] and in light of our findings, the EF-based classification of HF could be misleading, as it does not consider the pathophysiological mechanism and specific etiology underlying HF. Moreover, this classification is based on a parameter derived from a geometrical assumption, obtained by the modified Simpson’s rule, which is limited by suboptimal reproducibility [40]. In addition, EF is a load-dependent measure, which is strongly influenced by the patient’s hemodynamic state. With this regard, a patient with hypertrophic cardiomyopathy and concomitant hypovolemia may show a “supra-normal” EF; in this case, given the underlying severe diastolic dysfunction, despite an EF ≥ 65%, this patient has a low cardiac output, and his prognosis is worsened by the occurrence of a renal impairment and respiratory or non-respiratory infections. As a consequence, HFpEF patients with higher EF may have an unfavorable clinical course, similar to HFrEF patients, with increased mortality and hospitalization.

A number of limitations of the present study should be acknowledged. Firstly, it was a retrospective monocentric study that evaluated a limited number of hospitalized HFpEF patients. Secondly, to quantify the patient’s frailty, we used the AGILE score, a rapid and effective tool for screening multidimensional frailty more accessible to internal medicine physicians and cardiologists, rather than the multidimensional prognostic index (MPI), which is the gold standard for the assessment of elderly patients [41]. Third, systolic function was assessed by determining EF at hospital admission only, without measuring LV global longitudinal strain (GLS) by strain echocardiographic imaging and without collecting echocardiographic data at the time of discharge. Therefore, HFpEF diagnosis was only based on a single time-point EF measurement. Moreover, stroke volume and cardiac output were not calculated in our cohort of HFpEF patients; these parameters would have provided more information on the cardiac performance and hemodynamic status of HFpEF patients with different LVEF levels. Finally, cases of cardiac amyloidosis were not investigated in the present study, even if it is likely that, among HFpEF patients with hypertrophic cardiomyopathy, a fair number of patients could be affected by senile systemic amyloidosis.

## 5. Conclusions

Elderly HFpEF patients aged ≥ 70 y with “supra-normal” EF (≥65%) have a worse outcome than those with “normal” EF (50 to 64%) over a medium-term follow-up.

“Supra-normal” EF could be a surrogate indicator of hypovolemia, with important worse prognostic implications in frail elderly HFpEF patients.

A reclassification of HFpEF patients based on the recognition of HF with “normal” (between 50 and 64%) or “supra-normal” (≥65%) EF is needed, for a better prognostic risk stratification of these patients.

## Figures and Tables

**Table 1:** Baseline clinical characteristics of the whole HFpEF study population and of the two EF groups.

Baseline Clinical Parameters	All Patients (*n* = 200)	“Normal” EF (50–64%) (*n* = 99)	“Supra-Normal” EF (≥65%) (*n* = 101)	*p*-Value
**Demographics**				
Age (y)	86.4 ± 6.6	84.9 ± 6.8	87.7 ± 6.1	**0.002**
Age 70–84 y (*n*, %)	86 (43.0)	55 (55.5)	34 (33.7)	**<0.001**
Age ≥85 y (*n*, %)	114 (57.0)	44 (44.5)	70 (66.3)	**<0.001**
Female sex (*n*, %)	140 (70.0)	64 (64.6)	76 (75.2)	0.10
Male sex (*n*, %)	60 (30.0)	35 (35.4)	25 (24.8)	0.10
**Cardiovascular risk factors and comorbidities**				
Hypertension (*n*, %)	152 (76.0)	62 (62.6)	90 (89.1)	**<0.001**
Smoking (*n*, %)	32 (16.0)	19 (19.2)	13 (12.9)	0.22
Type 2 diabetes mellitus (*n*, %)	57 (28.5)	26 (26.3)	31 (30.7)	0.49
Dyslipidemia (*n*, %)	99 (49.5)	54 (54.5)	45 (45.4)	0.16
Anemia (Hb < 12 F or 13 g/dl M) (*n*, %)	124 (62.0)	58 (58.6)	66 (66.6)	0.32
CKD (eGFR < 60 mL/min/m^2^) (*n*, %)	119 (59.5)	57 (57.6)	79 (78.2)	**0.002**
COPD (*n*, %)	43 (21.5)	23 (23.2)	20 (19.8)	0.55
OSAS (*n*, %)	9 (4.5)	6 (6.1)	3 (2.9)	0.29
Hypothyroidism (*n*, %)	37 (18.5)	12 (12.1)	25 (24.7)	**0.02**
History of CAD (*n*, %)	41 (20.5)	27 (27.3)	14 (13.9)	**0.02**
Previous stroke (*n*, %)	37 (18.5)	18 (18.2)	19 (18.8)	0.91
Peripheral arteriopathy (*n*, %)	48 (24.0)	20 (20.2)	28 (27.7)	0.21
Cognitive impairment (*n*, %)	72 (36.0)	35 (35.3)	37 (36.6)	0.85
**Clinical prognostic scores**				
H_2_FPEF score	5.1 ± 1.9	5.0 ± 1.9	5.2 ± 2.0	0.47
Charlson comorbidity index	8.2 ± 2.1	7.8 ± 1.9	8.6 ± 2.2	**0.006**
AGILE score	6.4 ± 0.5	6.1 ± 0.4	6.6 ± 0.5	**<0.001**
**Physical examination**				
Dyspnea (*n*, %)	94 (47.0)	49 (49.5)	45 (45.4)	0.48
Leg swelling (*n*, %)	39 (19.5)	16 (16.2)	23 (22.7)	0.24
SBP (mmHg)	130.0 ± 29.0	134.0 ± 24.9	125.1 ± 32.7	**0.03**
DBP (mmHg)	69.0 ± 14.4	71.8 ± 13.8	66.1 ± 14.8	**0.005**
Body temperature ≥37.5° (*n*, %)	71 (35.5)	15 (15.1)	56 (55.4)	**<0.001**
**Chest X-ray**				
Normal pattern (*n*, %)	84 (42.0)	41 (41.4)	43 (42.6)	0.98
Congestion (*n*, %)	68 (34.0)	50 (50.5)	18 (17.8)	**<0.001**
Pneumonia (*n*, %)	48 (24.0)	8 (8.1)	40 (39.6)	**<0.001**
**ECG parameters**				
AF (*n*, %)	59 (29.5)	32 (32.3)	27 (26.7)	0.39
HR (bpm)	78.5 ± 17.1	73.0 ± 14.2	84.0 ± 19.6	**<0.001**
LBBB (*n*, %)	10 (5.0)	5 (5.1)	5 (4.9)	0.97
**Biochemical parameters**				
Serum hemoglobin (g/dl)	10.9 ± 2.3	11.1 ± 2.4	10.7 ± 2.3	0.23
Serum WBCs (× 10^9^/L)	12.5 ± 7.5	11.3 ± 6.3	13.6 ± 6.9	**0.01**
PaO2 (mmHg)	80.5 ± 35.8	88.9 ± 44.0	71.8 ± 19.7	**<0.001**
PaCO2 (mmHg)	37.4 ± 10.8	36.1 ± 7.3	39.0 ± 13.7	0.07
Serum glucose (mg/dl)	139.9 ± 106.2	135.9 ± 88.3	144.0 ± 121.8	0.59
Serum creatinine (mg/dl)	1.71 ± 1.40	1.49 ± 1.39	1.93 ± 1.38	**0.02**
eGFR (ml/min/m^2^)	46.0 ± 26.2	52.3 ± 26.6	39.9 ± 24.5	**<0.001**
Serum sodium (mEq/l)	139.1 ± 7.9	139.4 ± 6.8	138.8 ± 8.9	0.59
Serum potassium (mEq/l)	4.1 ± 0.7	4.1 ± 0.6	4.2 ± 0.8	0.32
Serum uric acid (mg/dl)	7.9 ± 7.5	7.3 ± 3.3	8.5 ± 10.1	0.26
Serum HDL cholesterol (mg/dl)	38.4 ± 14.1	39.0 ± 12.7	37.8 ± 15.5	0.55
Serum LDL cholesterol (mg/dl)	82.1 ± 34.4	83.0 ± 33.8	81.2 ± 35.0	0.72
Serum TSH (uU/mL)	1.95 ± 2.76	1.49 ± 1.53	2.41 ± 3.53	**0.02**
Serum CRP (mg/dl)	7.6 ± 8.1	5.3 ± 8.2	9.8 ± 8.0	**<0.001**
Serum NT-proBNP (pg/mL)	3267 ± 4674	3070 ± 4442	3583 ± 5016	0.44
Serum HS troponin (ng/mL)	123 ± 257	100 ± 200	147 ± 304	0.19

Data are expressed as mean ± SD or as number (percentage). Significant *p*-values are in bold. AF, atrial fibrillation; CAD, coronary artery disease; CKD, chronic kidney disease; COPD, chronic obstructive pulmonary disease; CRP, C-reactive protein; DBP, diastolic blood pressure; eGFR, estimated glomerular filtration rate; EF, ejection fraction; Hb, hemoglobin; HFpEF, heart failure with preserved ejection fraction; HR, heart rate; HS, high-sensitivity; LBBB, left bundle branch block; LDL, low-density lipoprotein; NT-proBNP, N-terminal pro-brain natriuretic peptide; OSAS, obstructive sleep apnea syndrome; PaO2, partial pressure of oxygen in the arterial blood; PaCO2, partial pressure of carbon dioxide in the arterial blood; SBP, systolic blood pressure; TSH, thyroid stimulating hormone; WBCs, white blood cells.

**Table 2:** Conventional echo Doppler parameters obtained at hospital admission in the whole HFpEF study population and in the two EF groups.

Echo Doppler Parameters	All Patients (*n* = 200)	“Normal” EF (50–64%) (*n* = 99)	“Supra-Normal” EF (≥65%) (*n* = 101)	*p*-Value
IVS (mm)	13.7 ± 2.4	13.3 ± 2.4	14.1 ± 2.3	**0.01**
PW (mm)	10.4 ± 1.2	10.2 ± 1.1	10.6 ± 1.2	**0.01**
LVEDD (mm)	43.3 ± 5.9	44.2 ± 5.5	42.5 ± 6.2	**0.04**
RWT	0.49 ± 0.08	0.47 ± 0.07	0.51 ± 0.08	**<0.001**
LVEDV (ml)	62.8 ± 21.2	65.7 ± 21.5	57.4 ± 20.8	**0.006**
LVESV (ml)	22.9 ± 9.5	28.0 ± 9.7	17.9 ± 7.0	**<0.001**
EF (%)	63.5 ± 6.8	57.4 ± 3.4	68.9 ± 3.9	**<0.001**
E/A ratio *	0.91 ± 0.44	1.05 ± 0.48	0.76 ± 0.40	**<0.001**
E/average e’ ratio **	14.6 ± 6.2	17.4 ± 5.5	11.8 ± 5.5	**<0.001**
Systolic IV pressure gradient ≥15 mmHg (*n*, %)	64 (23.0)	6 (6.1)	58 (57.4)	**<0.001**
LA A-P diameter (mm)	46.0 ± 9.5	45.9 ± 10.4	46.0 ± 8.6	0.94
LA longitudinal diameter (mm)	57.1 ± 12.1	56.7 ± 12.3	57.5 ± 12.0	0.64
LAV (ml)	83.3 ± 32.5	82.8 ± 32.8	83.7 ± 32.4	0.84
RVIT (mm)	30.7 ± 7.0	32.0 ± 7.4	29.5 ± 6.3	**0.01**
Moderate-to-severe MR (*n*, %)	63 (31.5)	45 (45.4)	18 (17.8)	**<0.001**
Moderate-to-severe AR (*n*, %)	20 (10.0)	12 (12.1)	8 (7.9)	0.32
Moderate-to-severe AS (*n*, %)	25 (12.5)	10 (10.1)	15 (14.8)	0.31
Moderate-to-severe TR (*n*, %)	64 (32.0)	32 (32.3)	32 (31.7)	0.92
TRV (m/s)	3.03 ± 0.58	3.06 ± 0.57	3.01 ± 0.59	0.54
IVC (mm)	19.9 ± 7.4	20.0 ± 7.2	19.9 ± 7.6	0.92
TAPSE (mm)	24.3 ± 4.4	26.4 ± 4.7	22.2 ± 4.1	**<0.001**
sPAP (mmHg)	41.8 ± 17.5	42.5 ± 18.7	41.1 ± 16.3	0.57
TAPSE/sPAP ratio (mm/mmHg)	0.58 ± 0.26	0.62 ± 0.21	0.54 ± 0.25	**0.01**
Aortic root (mm)	34.1 ± 4.1	34.0 ± 4.0	34.1 ± 4.2	0.86
Ascending aorta (mm)	35.4 ± 4.6	35.6 ± 4.2	35.1 ± 4.9	0.44

Data are expressed as mean ± SD or as number (percentage). Significant *p*-values are in bold. A-P, antero-posterior; AR, aortic regurgitation; AS, aortic stenosis; EF, ejection fraction; HpEF, heart failure with preserved ejection fraction; IVC, inferior vena cava; IV, intraventricular; IVS, interventricular septum; LA, left atrial; LAV, left atrial volume; LVEDD, left ventricular end-diastolic diameter; LVEDV, left ventricular end-diastolic volume; LVESV, left ventricular end-systolic volume; MR, mitral regurgitation; PW, posterior wall; RVIT, right ventricular inflow tract; RWT, relative wall thickness; sPAP, systolic pulmonary artery pressure; TAPSE, tricuspid annular plane systolic excursion; TR, tricuspid regurgitation; TRV, tricuspid regurgitation velocity. * Measured only in patients in sinus rhythm; ** measured in all patients.

**Table 3:** Relevant HFpEF characteristics and hospitalization data in the whole study population and in the two EF groups.

HF Characteristics and Hospitalization Parameters	All Patients (*n* = 200)	“Normal” EF (50–64%) (*n* = 99)	“Supra-Normal” EF (≥65%) (*n* = 101)	*p*-Value
**NYHA functional class**				
Class III (*n*, %)	100 (50.0)	60 (60.6)	40 (39.6)	**0.003**
Class IV (*n*, %)	100 (50.0)	39 (39.4)	61 (60.4)	**0.003**
**Underlying cardiac disease**				
Acute/chronic CAD (*n*, %)	50 (25.0)	36 (36.4)	14 (13.9)	**<0.001**
Acute/chronic VHD (*n*, %)	108 (54.0)	67 (67.7)	41 (40.6)	**<0.001**
Hypertensive cardiomyopathy (*n*, %)	152 (76.0)	62 (62.6)	90 (89.1)	**<0.001**
Acute/chronic pulmonary hypertension (*n*, %)	95 (47.5)	45 (45.5)	50 (49.5)	0.57
**Reasons for hospitalizations**				
Congestive heart failure (*n*, %)	73 (36.5)	55 (55.5)	18 (17.8)	**<0.001**
Pneumonia/bronchitis/respiratory failure/PE (*n*, %)	70 (35.0)	15 (15.1)	55 (54.5)	**<0.001**
Infections (urinary tract, intestine, endocarditis) (*n*, %)	35 (17.5)	10 (10.1)	25 (24.7)	**0.006**
Gastro-intestinal disorders (*n*, %)	35 (17.5)	11 (11.1)	24 (23.8)	**0.02**
Severe anemia (Hb < 8 g/dl) (*n*, %)	29 (14.5)	9 (9.1)	20 (19.8)	**0.03**
Severe CKD (eGFR < 15 mL/min/m^2^) (*n*, %)	29 (14.5)	9 (9.1)	20 (19.8)	**0.03**
Cancers (*n*, %)	29 (14.5)	9 (9.1)	20 (19.8)	**0.03**
Hyponatremia (*n*, %)	43 (21.5)	15 (15.1)	28 (27.7)	**0.03**
Hypernatremia (*n*, %)	31 (15.5)	10 (10.1)	21 (20.8)	**0.04**
Neurological disorders (*n*, %)	24 (12.0)	6 (6.1)	18 (17.8)	**0.01**
≥2 reasons for hospitalizations (*n* %)	83 (41.5)	25 (25.2)	58 (57.4)	**<0.001**
**Discharge therapy**				
Antiplatelets (*n*, %)	74 (37.0)	35 (35.3)	39 (38.6)	0.63
Anticoagulants (*n*, %)	69 (34.5)	36 (36.4)	33 (32.7)	0.58
ACEIs/ARBs (*n*, %)	65 (32.5)	38 (38.4)	28 (27.7)	0.11
CCB (*n*, %)	51 (25.5)	26 (26.3)	25 (24.7)	0.81
BB (*n*, %)	115 (57.5)	46 (46.5)	69 (68.3)	**0.002**
Digoxin (*n*, %)	42 (21.0)	18 (18.2)	24 (23.8)	0.33
Loop diuretics (*n*, %)	88 (44.0)	64 (64.6)	24 (23.8)	**<0.001**
Aldosterone antagonists (*n*, %)	73 (36.5)	38 (38.4)	35 (34.6)	0.58
Statins (*n*, %)	62 (31.0)	32 (32.3)	30 (29.7)	0.69
Oral hypoglycemic agents (*n*, %)	20 (10.0)	13 (13.1)	7 (6.9)	0.14
Insulin (*n*, %)	25 (12.5)	9 (9.1)	16 (15.8)	0.15
**Length of hospital stay (days)**	11.7 ± 5.5	11.4 ± 5.8	12.0 ± 5.2	0.44

Data are expressed as mean ± SD or as number (percentage). Significant *p*-values are in bold. ACEIs, angiotensin-converting enzyme inhibitors; ARBs, angiotensin II receptor blockers; BB, beta blockers; CAD, coronary artery disease; CCB, calcium-channel blockers; CKD, chronic kidney disease; eGFR, estimated glomerular filtration rate; EF, ejection fraction; Hb, hemoglobin; HpEF, heart failure with preserved ejection fraction; NYHA, New York Heart Association; PE, pulmonary embolism; VHD, valvular heart disease.

**Table 4:** Temporal analysis of in-hospital deaths and of the rates of mortality and rehospitalization due to non-cardiovascular or cardiovascular causes at 3-month, 6-month, 12-month-, and 36-month follow-up, recorded in the whole study population and in the two EF groups.

Outcome	All Patients (*n* = 200)	“Normal” EF (50–64%) (*n* = 99)	“Supra-Normal” EF (≥65%) (*n* = 101)	*p*-Value
**Mortality** (*n*, %)	79 (39.5)	7 (7.1)	72 (71.3)	**<0.001**
In-hospital (*n*, %)	11 (5.5)	1 (1.0)	10 (9.9)	**0.006**
3-month (*n*, %)	21 (10.5)	1 (1.0)	20 (19.8)	**<0.001**
6-month (*n*, %)	15 (7.5)	1 (1.0)	14 (13.9)	**<0.001**
12-month (*n*, %)	13 (6.5)	1 (1.0)	12 (11.9)	**0.002**
36-month (*n*, %)	18 (9.0)	3 (3.0)	15 (14.8)	**0.003**
**Rehospitalization due to non-CV causes** (*n*, %)	59 (29.5)	6 (6.1)	53 (52.5)	**<0.001**
3-month (*n*, %)	16 (8.0)	1 (1.0)	15 (14.8)	**<0.001**
6-month (*n*, %)	13 (6.5)	2 (2.0)	11 (10.9)	**0.01**
12-month (*n*, %)	12 (6.0)	1 (1.0)	11 (10.9)	**0.003**
36-month (*n*, %)	18 (9.0)	2 (2.0)	16 (15.8)	**<0.001**
**Rehospitalization due to CV causes** (*n*, %)	14 (7.0)	7 (7.1)	7 (6.9)	0.97
3-month (*n*, %)	4 (2.0)	2 (2.0)	2 (2.0)	0.98
6-month (*n*, %)	3 (1.5)	2 (2.0)	1 (1.0)	0.55
12-month (*n*, %)	3 (1.5)	2 (2.0)	1 (1.0)	0.55
36-month (*n*, %)	4 (2.0)	2 (2.0)	2 (2.0)	0.98

Data are expressed as number (percentage). Significant *p*-values are in bold. CV, cardiovascular; EF, ejection fraction.

**Table 5:** Univariate and multivariate Cox regression analysis for prediction of the primary endpoint.

	Univariate Cox Regression Analysis			Multivariate Cox Regression Analysis		
Variables	HR	95% CI	*p*-Value	HR	95% CI	*p*-Value
Age (y)	1.18	1.13–1.23	**<0.001**	1.09	1.03–1.16	**0.002**
Female sex	0.21	0.13–0.34	**<0.001**	0.71	0.41–1.23	0.22
eGFR (ml/min/m^2^)	0.98	0.97–0.99	**<0.001**	0.99	0.98–1.01	0.85
H_2_FPEF score	1.06	0.95–1.19	0.28			
AGILE score	1.68	1.07–2.63	**0.02**	1.07	0.66–1.73	0.79
LVEF (%)	1.19	1.15–123	**<0.001**	1.08	1.03–1.14	**0.004**
E/average e’	1.01	0.97–1.04	0.74			
TAPSE/sPAP ratio (mm/mmHg)	0.01	0.00–0.02	**<0.001**	0.14	0.03–0.61	**0.009**
Infectious disease occurring during the baseline stay	39.7	16.9–93.5	**<0.001**	7.23	2.41–21.6	**<0.001**

Significant *p*-values are in bold. eGFR, estimated glomerular filtration rate; LVEF, left ventricular ejection fraction; sPAP, systolic pulmonary artery pressure; TAPSE, tricuspid annular plane systolic excursion.

**Table 6:** Univariate and multivariate Cox regression analysis for the prediction of the secondary endpoint.

	Univariate Cox Regression Analysis			Multivariate Cox Regression Analysis		
Variables	HR	95% CI	*p*-Value	HR	95% CI	*p*-Value
Age (y)	1.03	1.00–1.05	**0.04**	1.01	0.98–1.04	0.42
Female sex	0.82	0.59–1.15	0.25			
eGFR (ml/min/m^2^)	0.99	0.98–1.00	**0.003**	0.99	0.98–1.00	0.27
AF	0.90	0.63–1.29	0.56			
Heart rate (bpm)	1.00	0.99–1.01	0.65			
H_2_FPEF score	1.02	0.94–1.10	0.66			
AGILE score	1.40	1.01–1.94	**0.04**	1.22	0.87–1.71	0.24
RWT	1.15	0.85–1.56	0.36			
LVEF (%)	1.07	1.05–1.10	**<0.001**	1.04	1.01–1.07	**0.02**
E/average e’	1.01	0.98–1.03	0.53			
TAPSE/sPAP ratio (mm/mmHg)	0.22	0.12–0.42	**<0.001**	0.56	0.25–1.26	0.16
Infectious disease occurring during the baseline stay	2.82	2.03–3.92	**<0.001**	1.51	0.89–2.56	0.12
Diuretics	1.16	0.81–1.64	0.41			
Length of hospital stay (days)	0.98	0.95–1.01	0.31			

Significant *p*-values are in bold. AF, atrial fibrillation; eGFR, estimated glomerular filtration rate; LVEF, left ventricular ejection fraction; RWT, relative wall thickness; sPAP, systolic pulmonary artery pressure; TAPSE, tricuspid annular plane systolic excursion.

## Data Availability

Data extracted from included studies will be publicly available on Zenodo (https://zenodo.org) (accessed on 3 January 2025).

## Supplementary Materials

The following supporting information can be downloaded at: https://www.mdpi.com/article/10.3390/jcm14020426/s1, Table S1: Demographic, clinical and instrumental data collected from patients’ hospital medical charts.
