# Supplementary material for: Supra-Normal Ejection Fraction at Hospital Admission Stratifies Mortality Risk in HFpEF Patients Aged ≥ 70 Years
Source: J Clin Med. 2025 Jan 10;14(2):426. doi: 10.3390/jcm14020426 (PMC11765728; doi:10.3390/jcm14020426)
Supplement: Supplementary file 1 [file jcm-14-00426-s001.zip › jcm-3426011-supplementary.pdf]

Table S1. Demographic, clinical and instrumental data collected from patients’ hospital medical charts.

Demographics: age, gender
Cardiovascular risk factors: hypertension, smoking, type 2 diabetes, dyslipidemia
Comorbidities: chronic kidney disease, history of coronary artery disease, peripheral arteriopathy, previous stroke and/or transient ischemic attack, cognitive impairment, chronic obstructive pulmonary disease, obstructive sleep apnea syndrome, hypothyroidism, anaemia
Blood tests: complete blood count, serum creatinine and estimated glomerular filtration rate, serum levels of glucose, sodium, potassium, uric acid, calcium, total bilirubin, cholesterol, thyroid-stimulating hormone, C-reactive protein, N-terminal pro-brain natriuretic peptide, high-sensitivity troponine
Blood pressure measurements
Electrocardiographic data (heart rate and cardiac rhythm)
Chest X-ray results
Current medical treatment
